# Downregulation of sphingosine kinase-1 induces protective tumor immunity by promoting M1 macrophage response in melanoma

**DOI:** 10.18632/oncotarget.12380

**Published:** 2016-09-30

**Authors:** Marguerite Mrad, Caroline Imbert, Virginie Garcia, Florian Rambow, Nicole Therville, Stéphane Carpentier, Bruno Ségui, Thierry Levade, Rania Azar, Jean-Christophe Marine, Mona Diab-Assaf, Céline Colacios, Nathalie Andrieu-Abadie

**Affiliations:** ^1^ Université de Toulouse, Equipe Labellisée Ligue Contre le Cancer 2013, Toulouse, France; ^2^ Inserm 1037, Centre de Recherches en Cancérologie de Toulouse, Equipe Labellisée Ligue Contre le Cancer 2013, Toulouse, France; ^3^ Molecular Tumorigenesis and Anticancer Pharmacology, EDST, Lebanese University, Hadath, Lebanon; ^4^ VIB, Center for the Biology of Disease, Leuven, Belgium; ^5^ Laboratoire de Biochimie Métabolique, Centre Hospitalier Universitaire Toulouse, Toulouse, France

**Keywords:** inflammation, melanoma, macrophage, polarization, sphingosine 1-phosphate

## Abstract

The infiltration of melanoma tumors by macrophages is often correlated with poor prognosis. However, the molecular signals that regulate the dialogue between malignant cells and the inflammatory microenvironment remain poorly understood. We previously reported an increased expression of sphingosine kinase-1 (SK1), which produces the bioactive lipid sphingosine 1-phosphate (S1P), in melanoma. The present study aimed at defining the role of tumor SK1 in the recruitment and differentiation of macrophages in melanoma. Herein, we show that downregulation of SK1 in melanoma cells causes a reduction in the percentage of CD206^high^MHCII^low^ M2 macrophages in favor of an increased proportion of CD206^low^MHCII^high^ M1 macrophages into the tumor. This macrophage differentiation orchestrates T lymphocyte recruitment as well as tumor rejection through the expression of Th1 cytokines and chemokines. *In vitro* experiments indicated that macrophage migration is triggered by the binding of tumor S1P to S1PR1 receptors present on macrophages whereas macrophage differentiation is stimulated by SK1-induced secretion of TGF-β1. Finally, RNA-seq analysis of human melanoma tumors revealed a positive correlation between SK1 and TGF-β1 expression. Altogether, our findings demonstrate that melanoma SK1 plays a key role in the recruitment and phenotypic shift of the tumor macrophages that promote melanoma growth.

## INTRODUCTION

Beside the cancer cells and their surrounding stroma, the tumor microenvironment contains innate and adaptive immune cells that can recognize and destroy tumor cells. However, mounting evidence indicates that tumor cells are able to change the functions of infiltrated immune cells to generate favorable conditions essential for tumor growth and progression [1].

In melanomas, macrophages represent a prominent component of the leukocytic infiltrate [2], and a high density of melanoma-associated macrophages correlates with poor clinical outcome [3]. Monocyte-derived macrophages are generally recruited from the blood to the tumor site by a wide array of biologically active molecules produced by malignant and stromal cells [4]. Infltrating macrophages respond to this milieu and can polarize, similarly to the CD4^+^ Th1 versus Th2 cell paradigm, into either M1 (classically activated) or M2 (alternatively activated) macrophages according to environmental stimuli [5]. Tumor-associated macrophages (TAMs) are key orchestrators of cancer-related inflammation and are considered to be of the M2 phenotype. These cells produce a plethora of growth factors, cytokines, chemokines, extracellular matrix proteins, and proteases, which promote tumor angiogenesis, growth, metastasis, and immune suppression [6, 7]. Indeed, TAMs affect adaptive immune responses by recruiting T regulatory cells, which in turn suppress antitumor effector cells such as NK cells and CD8+ T cells [8, 9].

One of the therapeutic approaches that provides clinically important benefit for patients with disseminated melanoma whose tumors contain the V600 mutation in the BRAF gene is based on the inhibition of the MAP kinase pathway with BRAF inhibitors. Interestingly, depletion of F4/80 and CD11b positive macrophages enhances the antitumor activity of BRAF inhibitors on mouse melanoma tumors. Conversely, the presence of macrophages in human tumors predicts early relapse after treatment [10], suggesting important reciprocal interactions between TAMs and malignant cells in melanoma tumors. However, the possible underlying mechanisms of these interactions are not fully understood and their possible therapeutic ramifications await further investigation.

Interestingly, we recently showed an increased production of sphingosine 1-phosphate (S1P) in melanoma cells [11, 12]. This bioactive sphingolipid metabolite is mainly produced by sphingosine kinase SK1, which is overexpressed in human melanoma tumors compared to nevi [12]. In many tumor models, S1P conveys oncogenic signals as an intracellular second messenger and/or through a family of G-protein coupled receptors (S1PR1-5) expressed both on cancer cells and their surrounding microenvironment [13, 14]. In melanoma tumors, dysregulation of S1P production in cancer cells elicits a fibrotic response in the tumor microenvironment, which in turn stimulates melanoma cell migration by promoting S1PR3 expression [12]. Additionally, treatment of mice with the S1P receptor modulator FTY720, which renders cells unresponsive to S1P activation by sequestering S1PR1 intracellularly, reduced melanoma progression by inhibiting tumor vascularization [15]. These findings illustrate the paracrine action of melanoma cells-exported S1P through S1PRs on tumor-stroma interactions. However, recent studies demonstrate that the SK1/S1P/S1PR axis plays an essential role in inflammation-associated cancer development [16]. Indeed, RNAi-based downregulation of SK1 or S1PR1 has been shown to block the persistent activation of the STAT3 transcription factor and the level of proinflammatory cytokines and reduce cancer progression in mouse models of inflammation [17, 18].

Therefore, the goal of this study was to investigate whether S1P produced by melanoma cells could control inflammation in these tumors as well as the antitumor immune response. Our present findings provide new insights into the role of SK1 in the recruitment and differentiation of macrophages and the adaptative immune response to control melanoma growth.

## RESULTS

### Downregulation of SK1 in melanoma cells reduces tumor growth and modifies the composition of tumor-infiltrating leukocytes

**Figure 1:**
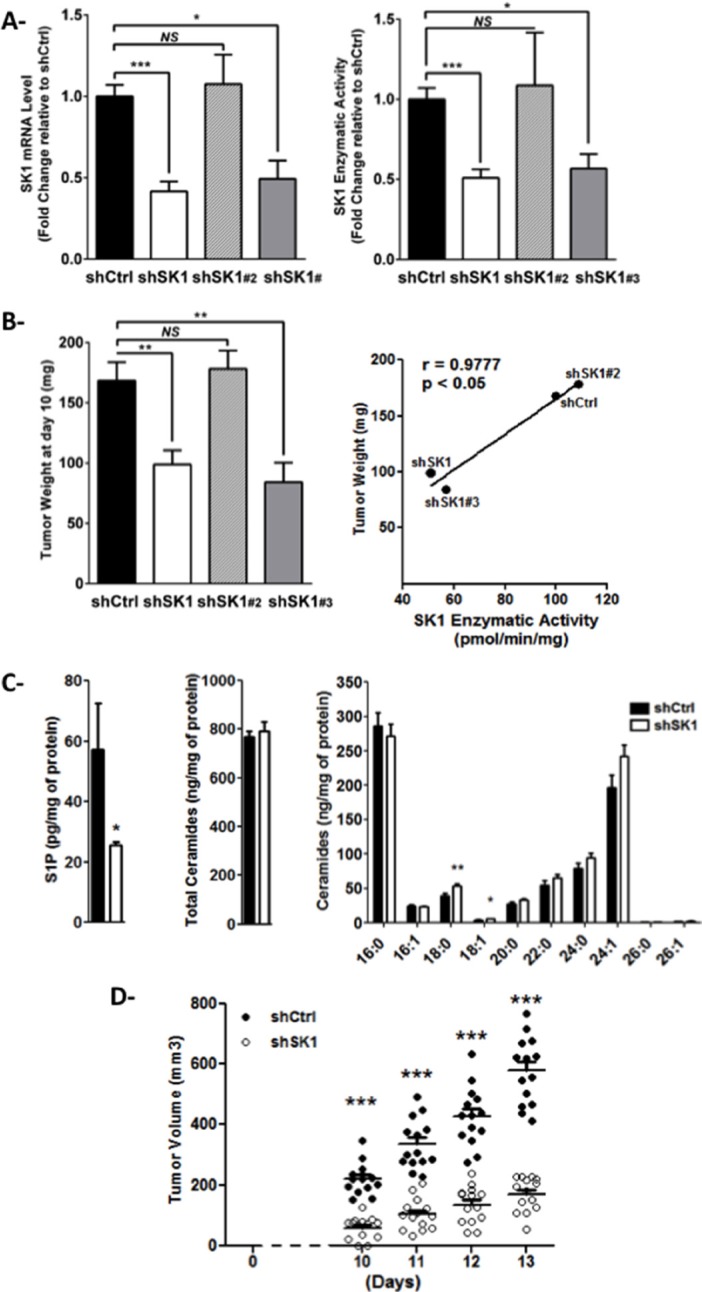
SK1 expression in melanoma cells drives tumor development in mice (**A**) SK1 mRNA level (left panel) and enzymatic activity (right panel) were measured in B16F10 cells stably transfected with a control (shCtrl) or SK1 targeted shRNA (shSK1, shSK1#2 or shSK1#3). Data are expressed as fold-change over shCtrl B16F10 cells and are means ± sem of 3–5 independent experiments. (**B**) B16F10 murine melanoma cells (3.10^5^) were injected in the dermis of C57BL/6 mice. After excision 10 days later, tumors were collected and weighed (left panel). Data are means ± sem (*n* = 4 to 5 mice per group). The relationship between SK1 enzymatic activity and tumor weight was evaluated with a Pearson correlation analysis (right panel). (**C**) Cellular lipids were extracted from shCtrl or shSK1 tumors and sphingolipid levels were quantified by LC/MS. Levels of S1P (left panel), total ceramide (middle panel) and individual ceramide species (right panel) were normalized to protein content. Results represent means ± sem of 2 independent experiments. (**D**) Tumor volume was determined at the indicated days in the mice that were implanted with shCtrl or shSK1 B16F10 cells, as described in B. Results are from 2 independent experiments performed with 7 mice per group. Values determined for individual tumors are depicted and horizontal lines correspond to means. For all panels, significant differences were evaluated using Student *t* test.

In order to evaluate the effect of SK1 in a syngeneic mouse model of melanoma, we first generated stable SK1 knockdown clones of B16F10 cells, using shRNA-mediated silencing technology. As shown in Figure 1A, two puromycin-resistant clones (shSK1 and shSK1#3) exhibited a markedly reduced mRNA expression as well as enzymatic activity of SK1, compared to B16 shCtrl. Expression of the SK2 isoenzyme was unchanged in SK1-downregulated cells (Supplementary Figure S1A). Importantly, B16 melanoma clones exhibited similar *in vitro* cell proliferation rates, irrespectively of SK1 mRNA level (Supplementary Figure S1B). Then, B16F10 cells knockdown or not for SK1 were intradermally injected in C57BL6 mice, and tumor weight was evaluated 10 days later. Figure 1B shows that, whereas the *in vivo* tumor growth of shSK1#2 B16F10 was not reduced compared to shCtrl B16F10, that of shSK1 and shSK1#3 B16F10 was significantly lower. The positive correlation found between SK1 activity and tumor weight highlights the tumor-promoting role of SK1 in melanoma.

A sphingolipidomic analysis showed that downregulation of SK1 in melanoma tumors significantly reduced the levels of S1P (Figure 1C, left) but did not alter the total ceramide content (Figure 1C, middle). However, the levels of C18:0 and C18:1 ceramide were modestly yet significantly increased (Figure 1C, right). The alteration in S1P content was associated with a decrease in tumor volume and growth (Figure 1D).

**Figure 2:**
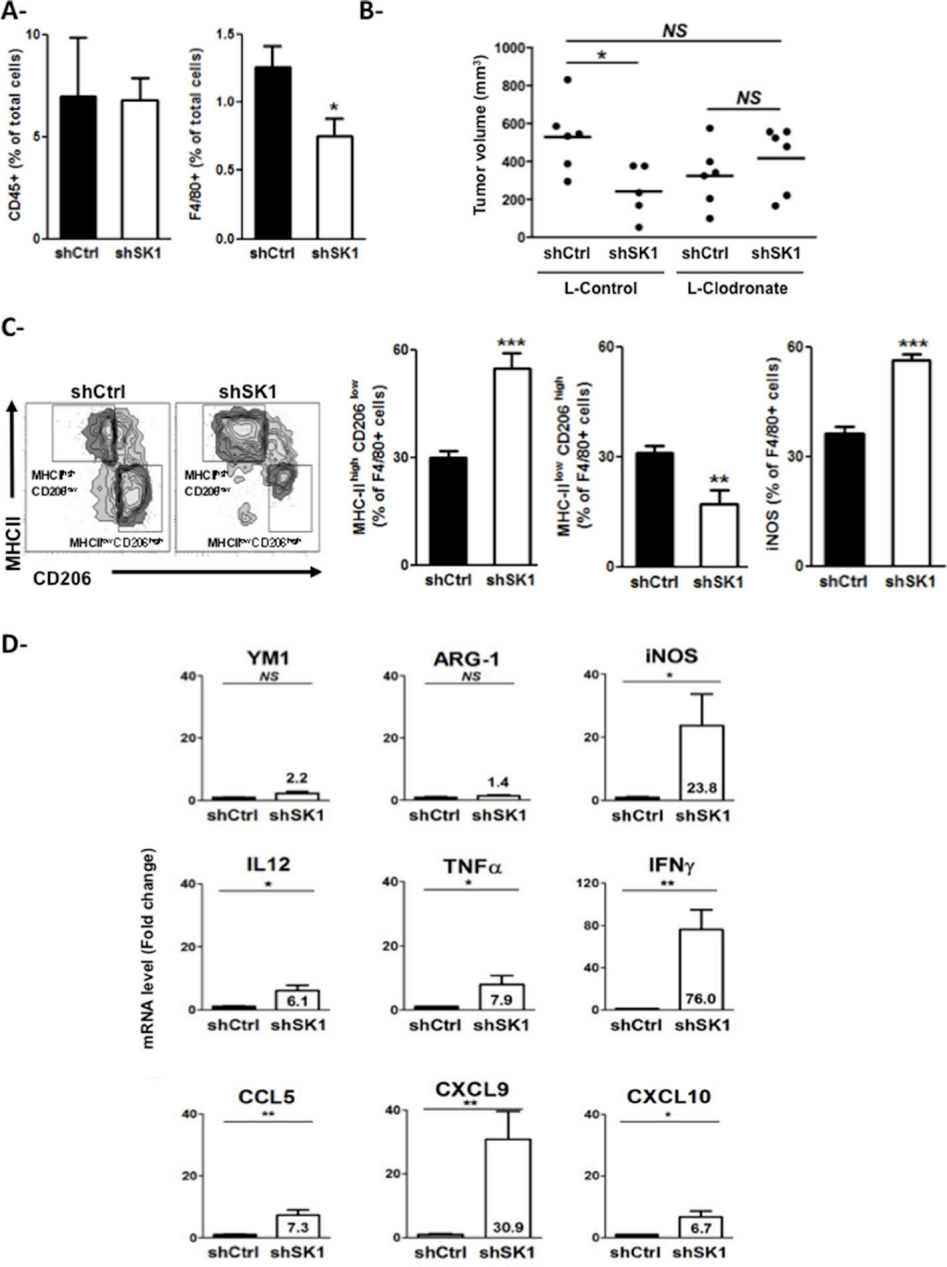
Expression of SK1 in melanoma cells alters the tumor infiltration and phenotype of macrophages (**A**, **C**) shCtrl or shSK1 B16F10 murine melanoma cells were injected in C57BL/6 mice. Ten days after injection, mice were sacrificed, tumors were collected, and their leukocyte content was analyzed. Bars represent means ± sem of 4 mice per group. Data are representative of two independent experiments. Significant differences were evaluated using Student *t* test. (A) Cells were counted and the percentage of CD45 and F4/80 among total cells was determined by flow cytometry. (B) Mice (*n* = 5–6/group) bearing shCtrl or shSK1 B16 tumors were treated with control liposomes (L-Control) or clodronate-containing liposomes (L-Clodronate) the day before tumor cell injection and then every 4 days for 2 weeks. Tumor volume was determined 13 days after melanoma cell implantation. Values determined for individual tumors are depicted and horizontal lines correspond to means. Data are representative of two independent experiments. Statistical analysis was performed using the Mann-Whitney *U*-test. (C) Representative flow cytometry density plots. Values indicate the percentages of MHC-II^high^ CD206^low^ (left panel), MHC-II^low^ CD206^high^ (middle panel) and iNOS^+^ (right panel) cells among the F4/80^+^ cells. (**D)** Ten days after injection, tumors were collected for mRNA isolation and analysis. Relative mRNA expression (fold induction relative to shCtrl) is depicted for M2 markers (YM1 and ARG-1) and M1 markers (Il12, Tnfα, Ifnγ, Ccl5, Cxcl9, Cxcl10). Significant differences were evaluated using Student *t* test. *NS*: not significant.

Then, to study whether and how SK1 could control inflammation in melanoma tumors, the leukocyte content of tumors, in which SK1 was reduced, was analyzed by flow cytometry and compared to that of control tumors. On day 10 after B16F10 inoculation, downregulation of SK1 did not affect the percentage of CD45^+^ leukocytes among the total cells collected from dissociated tumors but led to a 40% decrease in F4/80^+^ macrophage infiltration compared to the control (Figure 2A). Importantly, treatment of shSK1 B16 tumor-bearing mice with liposome encapsulated clodronate, a macrophage-depleting agent, abrogated tumor growth reduction induced by SK1 knockdown, suggesting that this effect is dependent on macrophages (Figure 2B). Moreover, the expression of cell surface markers such as the major histocompatibility complex class II (MHC-II) molecules, which are highly expressed on M1 macrophages, and the mannose receptor CD206, which is a specific marker of M2 macrophages, was investigated. As illustrated in Figure 2C, downregulation of SK1 in B16 increased the percentage of M1-oriented MHC-II^high^CD206^low^ TAMs (Figure 2C, left). In accordance, the tumor content of inducible nitric oxide synthase (iNOS^+^) F4/80^+^ cells (Figure 2C, right), which represent M1 macrophages, was higher in shSK1 B16 tumors than in shCtrl B16 tumors. In contrast, the percentage of M2-oriented MHC-II^low^CD206^high^ TAMs was decreased in melanoma tumors in which SK1 was inhibited (Figure 2C, middle).

In addition, downregulation of SK1 in B16 led to an increased expression of Th1 cytokines (IL-12, TNFa, IFNg) and chemokines (CCL5, CXCL9, CXCL10) into the tumor. In accordance, whereas mRNA expression of iNOS was significantly higher in shSK1 B16 tumors compared to that of shCtrl B16 tumors, the expression of the M2 markers YM1 and arginase-1 (ARG-1) was unchanged (Figure 2D).

Because M1 macrophages process tumor antigens and present them to T lymphocytes, which become activated, proliferate, and infiltrate the tumor [19], we next investigated the percentage of T lymphocytes in melanoma tumors. Interestingly, the proportion of different lymphocyte subpopulations was altered in tumors in which SK1 was inhibited, with a significant increase of NK cells as well as T-cells (Thy1^+^) such as CD4^+^ and CD8^+^ tumor-infiltrating lymphocytes (Supplementary Figure S2). In contrast, the percentage of B lymphocytes was decreased in tumors in which SK1 was reduced. In addition, the proportion of intratumor CD11b^+^Gr1^+^ myeloid suppressor-type cells was not modified by SK1 downregulation.

Collectively, our data indicate that melanoma SK1/S1P can control tumor growth by regulating the migration and polarization of macrophages.

### Downregulation of SK1 in melanoma cells impairs the migration of bone marrow-derived macrophages via S1PRs

**Figure 3:**
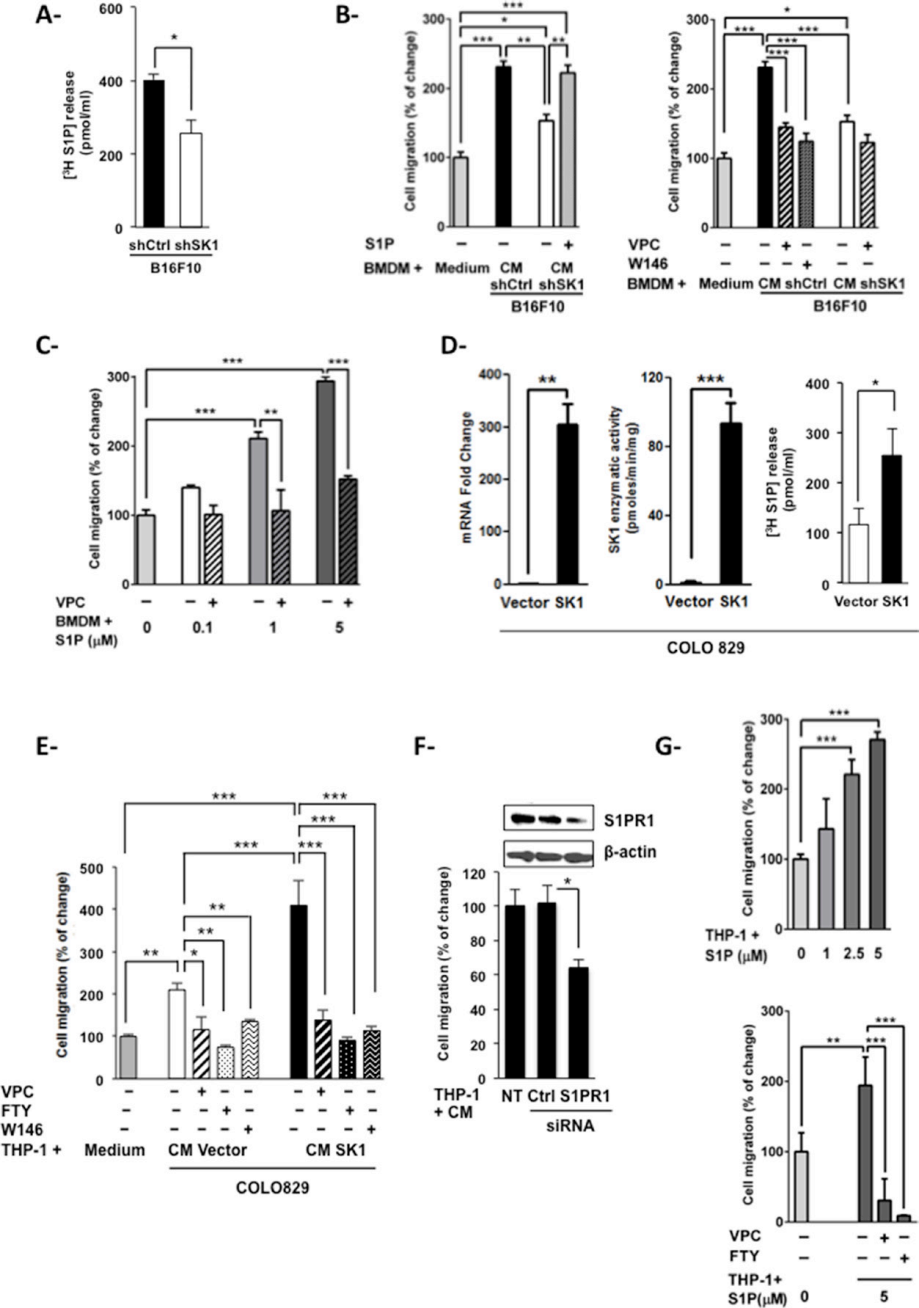
Macrophage migration is increased by melanoma-derived S1P (**A**) S1P release from shCtrl or shSK1 B16F10 melanoma cells as determined after the conversion of [^3^H]sphingosine to [^3^H]S1P. Concentrations of radiolabeled S1P in the medium are expressed as mean ± sem of three independent experiments. Significant differences were evaluated using Student *t* test. Transwell migration assays were performed to evaluate migration of BMDM (**B** and **C**) or THP-1 cells (**E**–**G**). Data are expressed as percent increase or decrease over migration in serum-free medium, and are means ± sem of 2–4 independent experiments. For panels B, C and E-G, significant differences were evaluated using one-way ANOVA with post hoc Tukey test. (B and C) BMDM were pre-treated (+) or not (−) with 2 μM VPC23019 (VPC) or 5 μM W146 for 1 hour. Then, BMDM were incubated for 5 hours in serum-free medium alone (Medium), the conditioned medium (CM) from shCtrl or shSK1 B16F10 melanoma cells containing or not S1P (B) or in serum-free medium containing S1P at the indicated concentrations (C). (**D**) SK1 mRNA expression (left panel), enzymatic activity (middle panel) and S1P release (right panel) were measured in COLO829 melanoma cells transfected either with an empty vector (Vector) or a plasmid encoding human SK1 (SK1). Data are means ± sem of 3 independent experiments. Significant differences were evaluated using Student *t* test. E and G, THP-1 cells were pre-treated or not with 2 μM VPC23019, 2 μM FTY720 (FTY) or 5 μM W146 for 1 hour. Then, THP-1 cells were incubated for 6 hours in the CM from control (Vector) or SK1-overexpressing (SK1) COLO829 melanoma cells (E) or in serum-free medium containing S1P at the indicated concentrations (G). F, Melanoma CM-induced cell migration was evaluated, as described in E, on THP-1 cells 48 hours after transfection with control (Ctrl) or S1PR1 siRNA (20 nM). Silencing of S1PR1 in THP-1 cells by siRNA was assessed by Western blot.

To explore the chemotactic behavior of macrophages in response to the melanoma SK/S1P/S1PR signaling pathway, bone marrow-derived macrophages (BMDM) were incubated with the conditioned medium (CM) from shCtrl or shSK1 B16 cells, and analyzed for their ability to migrate toward factors secreted by melanoma cells. Results show that depletion of SK1 in B16 cells led to reduced release of S1P in the medium (Figure 3A). Moreover, whereas the CM from shCtrl B16 cells exerted a powerful pro-migratory effect on BMDM as compared to BMDM incubated in medium alone, this effect was strongly attenuated when BMDM were exposed to the CM from shSK1 B16 cells (Figure 3B, left). Importantly, BMDM migration was restored by adding exogenous S1P to the CM of shSK1 B16 cells. Since S1PR1, 2 and 3 are expressed by BMDM [20], we next examined whether S1PR1 and S1PR3, which are known to promote cell migration, are implicated in BMDM migration upon incubation with melanoma CM. Figure 3B (right) shows that pretreatment of BMDM with the S1PR1 antagonist W146 or the S1PR1/3 antagonist VPC23019 impaired the migratory effect exerted by the CM from shCtrl B16 cells. Moreover, the direct treatment of BMDM with exogenous S1P mimicked, in a dose-dependent manner, the macrophage migration induced by the CM from shCtrl B16 cells. Again, pretreatment with VPC23019 abrogated S1P-induced BMDM migration (Figure 3C).

To confirm that melanoma SK1 controls macrophage migration, we used the human melanoma cell line COLO829, which is devoid of SK1 protein [12], and generated the COLO829(SK1) variant, which exhibited a sharp increase in mRNA expression (Figure 3D, left) as well as enzymatic activity (Figure 3D, middle) of SK1. These modifications led to increased S1P release in the CM from melanoma cells (Figure 3D, right). Then, we analyzed the migration of the human monocytic cell line THP-1 upon incubation with the CM either from COLO829(SK1) or control COLO829. The results show that THP-1 cell migration was enhanced upon incubation with the medium from COLO829 cells, a phenomenon further augmented when melanoma cells overexpressed SK1 (Figure 3E). Pretreatment of THP-1 cells with VPC23019, W146 or the S1PR1 modulator FTY720 abrogated the migratory effect exerted by the CM from COLO829 cells (Figure 3E). A significant reduction in macrophage migration was also obtained when THP-1 cells were transiently transfected with siRNA against S1PR1 (Figure 3F). Moreover, S1P treatment of THP-1 cells mimicked the macrophage migration induced by the CM from melanoma cells. Again, inhibitors targeting S1PR1 abrogated S1P-induced macrophage migration (Figure 3G).

Altogether, these results suggest that S1P produced by melanoma cells stimulates macrophage migration in a S1PR1-dependent manner.

### Downregulation of SK1 in melanoma cells induces the expression of M1 markers in bone marrow-derived macrophages

**Figure 4:**
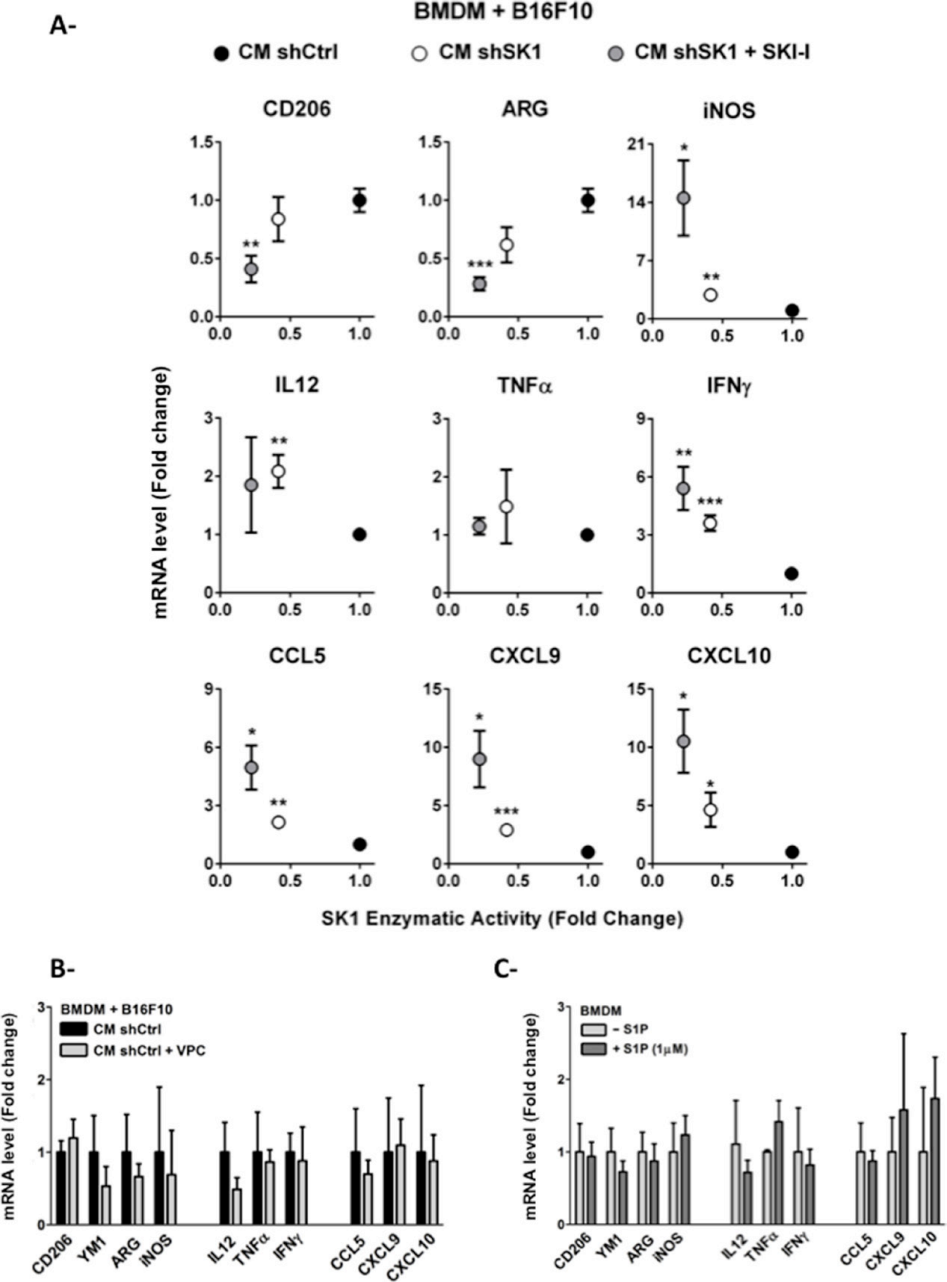
Macrophage polarization is controlled by melanoma SK1 through a S1PR-independent mechanism BMDM were incubated for 24 hours in the conditioned medium (CM) from B16F10 melanoma cells (**A** and **B**) or serum-free medium containing or not 1 μM S1P (**C**). Relative mRNA expression is depicted for M2 markers (*Mrc1*(CD206), *Chi3l3* (YM1) *and Arg1*) and M1 markers (*iNos*, *Il12*, *Tnfα*, *Ifnγ*, *Ccl5*, *Cxcl9*, *Cxcl10*). Data are plotted against the SK1 activity of B16F10 melanoma cells submitted to distinct treatments. (A) CM was prepared from shCtrl or shSK1 B16F10 melanoma cells treated or not with 3 μM SKI-I for 48 hours. (B) BMDM were pre-treated with 2 μM VPC23019 before incubation with the CM from shCtrl B16F10 melanoma cells. Data are expressed as fold-increase over shCtrl B16F10 cells and are means ± sem of 2–6 independent experiments. (C) Data are expressed as fold-change over migration in serum-free medium, and are means ± sem of 3–4 independent experiments. For all panels, significant differences were evaluated using Student *t* test.

**Figure 5:**
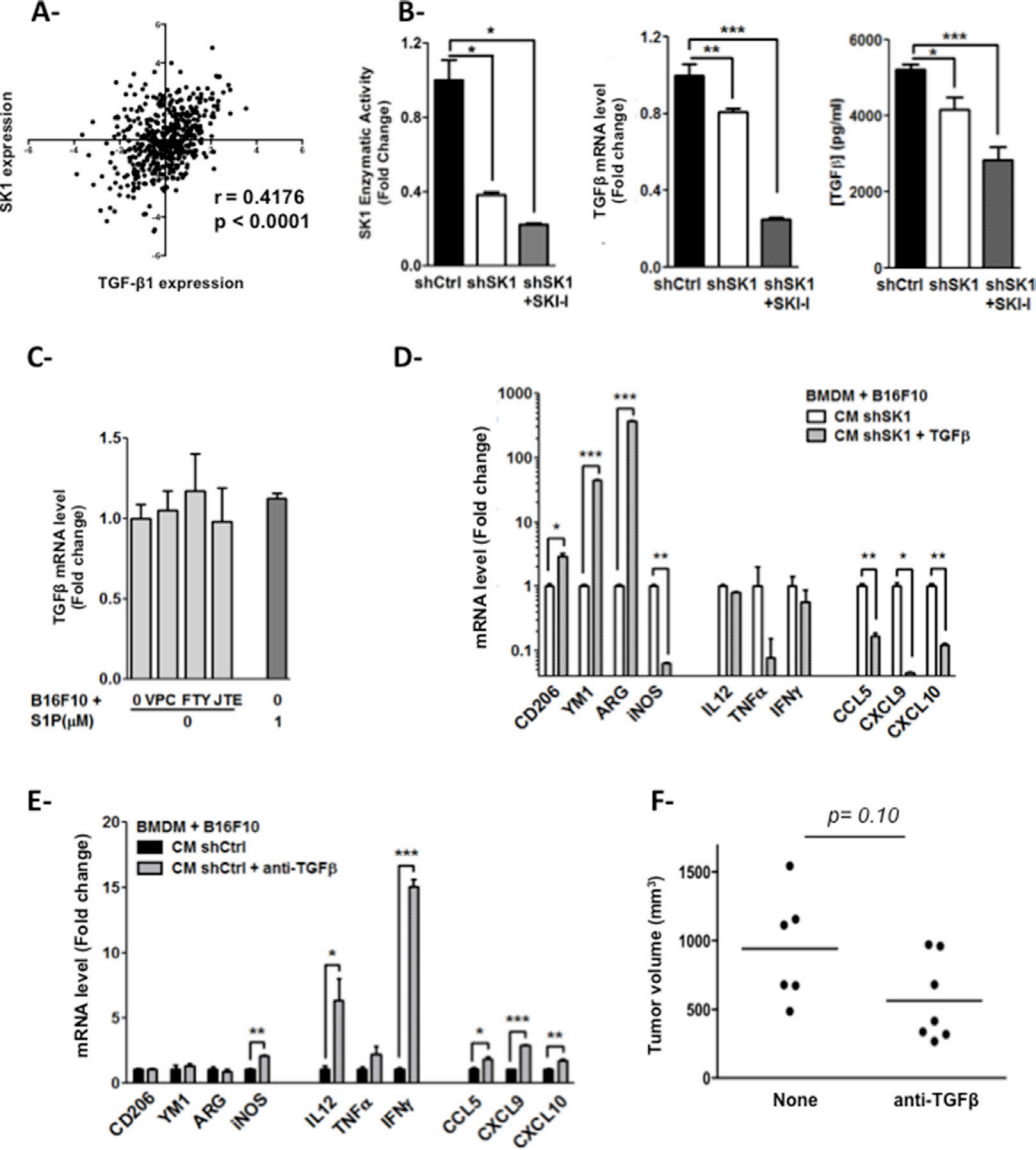
Melanoma SK1-induced TGF-β1 stimulates macrophage polarization (**A**) Analysis of SK1 and TGF-β1 expression in melanoma tumors was carried out using the TCGA database. (**B**) SK1 enzymatic activity (left), TGF-β1 mRNA expression (middle) and TGF-β1 secreted protein level (right) were measured in shCtrl or shSK1 B16F10 melanoma cells treated or not with 3 μM SKI-I for 48 hours. (**C**) TGF-β1 mRNA level was measured in shCtrl B16F10 melanoma cells pretreated or not with 2 μM VPC23019, 2 μM FTY720 or 10 μM JTE013 and incubated with 1 μM S1P for 24 hours. Data are expressed as fold-increase over shCtrl B16F10 cells and are means ± sem of 2–3 independent experiments. Significant differences were evaluated using Student *t* test. (**D** and **E**) BMDM were incubated for 48 hours in the conditioned medium (CM) from shCtrl or shSK1 B16F10 melanoma cells. Relative mRNA level is depicted for M2 markers (CD206, YM1 and Arg-1) and M1 markers (iNos, Il12, Tnfα, Ifnγ, Ccl5, Cxcl9, Cxcl10). D, Recombinant murine TGF-β1 (50 ng/ml) was added to the CM from shSK1 B16F10 melanoma cells before incubation with BMDM. E, Anti-TGF-β1 (1 μg/ml) was added to the CM from shCtrl B16F10 melanoma cells before incubation with BMDM. Results represent means ± sem of 2–3 independent experiments. Significant differences were evaluated using Student *t* test. (**F**) Mice (*n* = 6/group) bearing shCtrl B16 tumors were treated with TGF-b-neutralizing antibody or PBS, one day after tumor cell injection and then three times per week for 2 weeks. Tumor volume was determined 13 days after implantation. Values determined for individual tumors are depicted and horizontal lines correspond to means. Data are representative of two independent experiments. Statistical analysis was performed using the Mann-Whitney *U*-test.

Because an increased number of M1-oriented MHC-II^high^CD206^−^ TAMs were present in shSK1 melanoma tumors (see Figure 2C), we next studied the effect of SK1 inhibition on macrophage polarization *in vitro*. To this end, BMDM were incubated with the CM of B16 melanoma cells and the expression of M1 and M2 markers was analyzed. As shown in Figure 4, whereas downregulation of SK1 in shSK1 B16 cells significantly increased the expression of iNOS in BMDM, it also led to a strong decrease of ARG-1 expression. Moreover, the expression of Th1 cytokines and chemokines was increased in BMDM upon incubation with CM from shSK1 B16 cells as compared to those treated with CM from shCtrl B16 cells. Similar results were obtained when B16 cells were exposed to SKI-I, a pharmacological inhibitor of SK1 (data not shown). Interestingly, for most M1 markers, a further increase in the mRNA expression was observed (Figure 4A) when shSK1 B16 cells were pre-treated with SKI-I, which led to further inhibition of SK1 enzymatic activity (Figure 5B, left). These results demonstrate an inverse correlation between melanoma SK1 enzymatic activity and the expression of M1 markers in BMDM. Conversely, a positive corelation was found between SK1 activity and the expression of the M2 markers, CD206 and ARG-1 (Figure 4A) but not for YM1 (data not shown).

To determine whether these effects could be related to S1P secretion from melanoma cells, BMDM were preincubated or not with VPC23019 before the addition of the CM from shCtrl B16 cells. Figure 4B shows that the expression of M1 and M2 markers was not significantly modified by S1PR antagonism. Accordingly, direct treatment of BMDM with 1 μM (Figure 4C) or 5 μM (data not shown) S1P did not induce any variations in macrophage differentiation as compared to BMDM incubated in medium without S1P.

Altogether, these findings indicate that macrophage polarization induced by melanoma SK1 occurs through a S1P/S1PR-independent way.

### SK1-induced secretion of TGF-β1 stimulates macrophage polarization

To study further on the molecular mechanisms that control macrophage polarization, we analyzed an RNA-seq dataset obtained from a collection of 472 human cutaneous melanoma tissue samples (TCGA). We observed that the expression of the immunosuppressive cytokine TGF-β1 was slightly, albeit significantly, lower in samples displaying a lower SK1 expression (Figure 5A; *p* < 0.0001), supporting a positive correlation between SK1 and TGF-β1 expression in human melanoma tumors. Moreover, given that SK1 expression has been reported to correlate with the levels of TGF-β1 in different inflammation-based experimental models [21, 22], we next explored whether the effects of melanoma SK1 on macrophage polarization could be mediated by TGF-β1. Quantification of relative mRNA expression (Figure 5B, middle) as well as ELISA performed on cell-free supernatants (Figure 5B, right), revealed that downregulation of SK1 (Figure 5B, left) is associated with a significant decrease in TGF-β1 levels in melanoma cells, this phenomenom being amplified by SKI-I.

As most of the SK1/S1P-dependent immuno-modulatory effects are attributed to S1PR ligation, we next investigated the role of S1PRs in the regulation of SK1-induced TGF-β1 gene expression. Figure 5C shows that inhibition of S1PRs with three different antagonists, including the S1PR2-specific antagonist JTE013, or the direct treatment of B16F10 with exogenous S1P did not significantly change the TGF-β1 transcript levels. These results suggest that SK1-induced TGF-β1 gene expression is independent of the S1P/S1PR signaling pathways in melanoma cells.

Interestingly, addition of a recombinant TGF-β1 protein to the CM of shSK1 B16F10 rescued the ability of BMDM to polarize toward an M2 phenotype in response to melanoma cell signals. Moreover, under these conditions, the expression of M1 markers was reduced (Figure 5D). Reciprocally, addition of an anti-TGF-β neutralizing antibody to the CM shCtrl B16F10 increased the expression of M1 markers in BMDM (Figure 5E). This effect mimicked the response observed in BMDM incubated with the CM from shSK1 B16F10 (Figure 4A). Finally, treatment of shCtrlB16 tumor-bearing mice with an anti-TGF-β resulted in a tendency to tumor regression (Figure 5F). These findings indicate that melanoma SK1 stimulates macrophage differentiation as well as tumor growth through TGF-β1 production.

## DISCUSSION

Inflammation has long been associated with the development of cancer. A common feature of melanoma is the infiltration of immature macrophages at early stages of tumorigenesis. These cells modulate the activity of T lymphocytes and stroma cells, either promoting or inhibiting tumor progression depending on TAM infiltration density and differentiation status [23]. Accordingly, it has been shown in different murine tumor models that either depletion of macrophages or switching the phenotype of these cells into tumor-fighting M1 macrophages can significantly halt tumor growth [24, 25].

**Figure 6:**
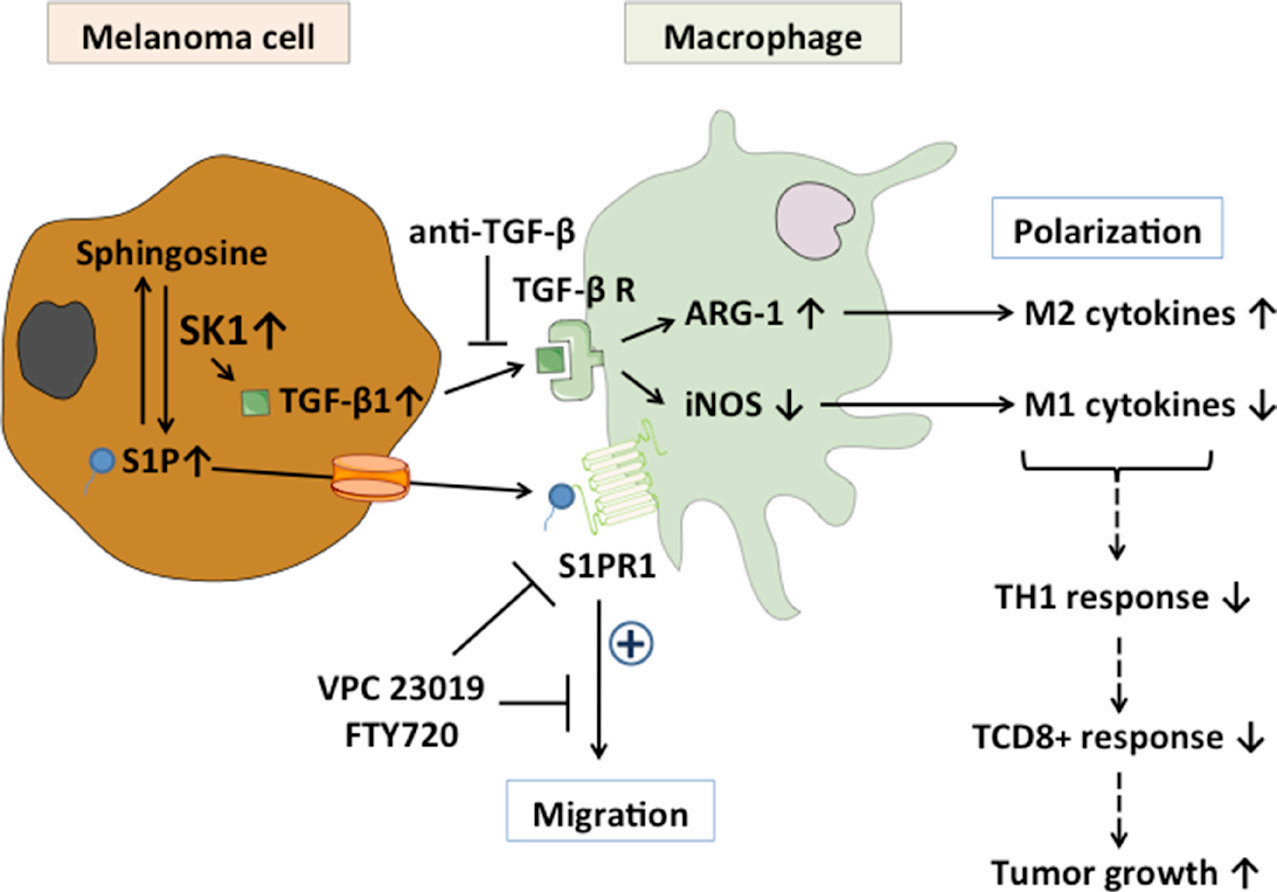
Role of melanoma SK1/S1P on macrophage migration and polarization Expression of TGF-β1 in melanoma cells is activated by the SK1/S1P pathway Co-incubation of macrophages with melanoma cells leads to increased ARG-1 expression and, inversely, decreased iNOS expression. This is associated with increased expression of M2-related cytokines and, inversely, decreased expression of M1-related cytokines as well as TCD8+ response, leading to increased tumor growth. Whereas blocking TGF-β1 with an anti-TGF-β neutralizing antibody abolished macrophage polarization towards an M2 phenotype, antagonism of S1PR1 inhibited macrophage migration induced by S1P.

Here, we report for the first time that SK1 regulates melanoma growth by modulating the macrophage infiltration of the tumor (see Figure 6). Indeed, the percentage of F4/80^+^ macrophages was lower in tumors in which SK1 was silenced than in control tumors. In accordance, a large increase of SK1 protein is associated with an increased infiltration of macrophages into tumor tissues of *Sphk2*^−/−^ mice developing colitis-associated cancer. In the latter model, SK1 and S1PR1 stimulate tumor growth and drive TAMs and dendritic cells to produce elevated IL-6 levels, thereby promoting a pro-inflammatory tumor microenvironment [18]. These observations suggest that SK1 could be involved in the migration/trafficking of macrophages leading to their infiltration of tumors. *In vitro*, our findings demonstrate that S1P produced by melanoma SK1 acts as a potent chemoattractant for macrophages in a S1PR1-dependent manner. Indeed, treatment of murine BMDM or the human cell line THP-1 with exogenous S1P promoted cell migration. Our results were obtained on THP-1 cells differentiated into macrophage-like cells by PMA, as under these conditions THP-1 cells recapitulate several features of human native monocyte-derived macrophages. THP-1 cells are widely used to investigate migration and differentiation of human macrophages in response to various inflammatory mediators. Here, we show that after treatment with S1P, THP-1 cells migrate as previously reported for human native monocyte-derived macrophages [26]. Whether S1P produced by melanoma SK1 stimulates macrophage infiltration through activation of S1PR *in vivo* remains to be determined. Nevertheless, in different experimental mouse models of inflammation, FTY720 reduced macrophage recruitment to the sites of inflammation, demonstrating that S1PR1 can be critical for macrophage trafficking *in vivo* [27, 28]. In contrast, downregulation in cancer cells of the SK2 isoform, which differs from SK1 by its subcellular localization, failed to reduce the number of macrophages in breast tumors [29].

Our study also identified SK1 as a key regulator of macrophage polarization in melanoma tumors (see Figure 6). Indeed, whereas knockdown of SK1 significantly increased the percentage of MHC-II^high^CD206^low^ M1 macrophages into the tumor, it reduced the proportion of MHC-II^low^CD206^high^ M2 macrophages. Moreover, the ratio of iNOS to ARG-1 gene expression, which is commonly used as a readout of macrophage functional status, was also increased in shSK1 B16 tumors. M1 macrophages are potent tumor-fighting cells and are able to suppress M2-associated tumor-promoting functions by a poorly understood mechanism [30]. Through the expression and secretion of cytokines and chemokines such as IL-12, CXCL9 and CXCL10, M1 macrophages drive the polarization and recruitment of Th1 cells. Reciprocally, by producing IFNγ, Th1 cells can drive classical M1 polarization of macrophages, thereby amplifying a type 1 response [31]. Our *in vitro* findings demonstrate that melanoma SK1 enzymatic activity correlates with a decrease of M1 markers, and conversely, an increase of M2 markers in BMDM. Previous reports have suggested that stimulation of S1PR1 by S1P reduces the expression of iNOS, TNFa, MCP-1 and IL-12, and induces ARG-1 expression in LPS-treated macrophages [32]. S1P-induced M2 polarization also occurs through IL-4 secretion in LPS-treated mouse peritoneal macrophages [33]. Here, we show that the phenotypic switch of macrophages induced by melanoma SK1 does not rely on the binding of S1P to S1PRs on macrophages. Indeed, the expression of M1- and M2-specific markers was not modified in macrophages pretreated with VPC23019 as compared to that of macrophages incubated with CM alone. Furthermore, addition of exogenous S1P to BMDM failed to promote macrophage polarization.

Our observations clearly show that melanoma SK1 stimulates tumor-derived TGF-β1 secretion, which leads to the differentiation of macrophages poorly expressing M1 phenotype genes. Neutralization of TGF-β1 in the CM from control melanoma cells stimulated M1 markers in BMDM. TGF-β1 signaling has previously been reported to be activated following ligation of S1PR2 by S1P [34]. Here, we demonstrate that SK1-induced TGF-β1 mRNA expression is not mediated by an autocrine S1P signaling loop through S1PRs. However, given that SK1 has been shown to activate transcription factors such as HIF-1α [35] or NF-κB [36] in various tumors and that TGF-β1 expression is induced by these factors [37, 38], one can speculate that SK1 stimulates TGF-β1 expression at a transcriptional level. However, this hypothesis still requires further investigation. Melanoma produces increasing amounts of TGF-β1 with disease progression [39]. TGF-β1 is one of the most potent immunosuppressive cytokine secreted by tumor cells. Notably, TGF-β1 counteracts effector functions of macrophages, NK cells, cytotoxic T lymphocytes and dendritic cells as well as cytokine secretion [40]. On macrophages, binding of TGF-β1 to its receptor leads to activation and nuclear translocation of Smad molecules, which cooperate with other transcription factors to regulate gene expression and reprogramme macrophages to the M2 phenotype [41].

Finally, in a number of preclinical models, several TGF-β inhibitors, including small-molecule inhibitors targeting type I and II TGF-β receptor activity, monoclonal antibodies neutralizing TGF-β ligands and antisense oligonucleotides blocking TGF-β ligand production have demonstrated antitumor activity. For instance, in B16 murine melanoma, anti-TGF-β therapy in combination with interleukin-2 reduced the number of lung metastases [42]. In melanoma patients, the baseline serum TGF-β levels were significantly higher than those in the control group [43]. Recently, an antitumor activity has been described for fresolimumab, a human anti-TGF-β monoclonal neutralizing antibody, which was administered to patients with advanced malignant melanoma [44].

Collectively, our data identify a novel role for the S1P-producing enzyme SK1 as a key regulator of the balance between inflammatory and suppressive macrophages in melanoma tumors.

## MATERIALS AND METHODS

### Cell culture and generation of conditioned medium

Melanoma cell lines were obtained from ATCC and grown as monolayers in RPMI or DMEM media supplemented with 10% heat-inactivated fetal calf serum (FCS) in the presence of 5% CO_2_ in a humidified atmosphere at 37°C. To guarantee cell line authenticity, B16F10 and COLO829 cell lines were used for a limited number of passages and routinely tested for the expression of melanocyte-lineage proteins such as MelanA/MART1. Human monocytic THP-1 cells obtained from Dr. A. Coste (University of Toulouse, France) were cultured in RPMI containing 10% FCS and 0.02 mM β-mercaptoethanol. THP-1 cells were differentiated into macrophages by stimulation with 20 ng/ml PMA (Sigma) for 24 hours; then cells were cultured for an additional 24 hours without PMA.

Conditioned media (CM) were produced by culturing melanoma cells in serum-free RPMI for 48 hours. In some experiments, melanoma cells were treated with 3 μM sphingosine kinase inhibitor SKI-I (5-(2-Naphthalenyl)-1*H*-pyrazole-3-carboxylic acid 2-[(2-hydroxy-1-naphthalenyl)methylene]hydrazide; Abcam). At the end of the culture period, the CM were collected, centrifuged for 5 min at 1500 rpm and filtered. Macrophages were then treated with the CM alone or in the presence of 5 μM S1P, 50 ng/ml recombinant TGF-β1 (Ebioscience SAS, Paris, France) or 1 μg/ml TGF-β1 antibody (Clone #1D11, R&D systems, Lille, France) for 48 hours.

### Isolation and culture of BMDM

Bone marrow cells were isolated from femurs and tibias of 12-week-old C57BL/6 mice and cultured (1.3 × 10^7^/dish; Bioscience Inc) in RPMI containing 10% FCS and 20 ng/ml recombinant murine macrophage colony-stimulating factor (rmM-CSF; Immunotools). After 7 days, adherent BMDM were harvested, counted and incubated or not with CM from melanoma cells.

### Cell transfection

B16F10 cells were co-transfected, in a 1:10 ratio, with the pEGFP-C1 empty vector plus one SK1 shRNA plasmid (shSK1 B16F10, 3 different shRNA from Thermoscientific were used) or a control (shCtrl B16F10) non-targeting shRNA plasmid (pLK01, Addgene). The hairpin sequences were:

5′-CCGG-GCACCCAAACTACCTTTGGAT-CTCGAG-ATCCAAAGGTAGTTTGGGTGC-TTTTT-3′ for shSK1, 5′-CCGG-GAGGCAGAGATAACCTTTAAA-CTCGAG-TTTAAAGGTTATCTCTGCCTC-TTTTT-3′ for shSK1#2 and 5′-CCGG-GCAGGTGACTAATGAAGACCT-CTCGAG-AGGTCTTCATTAGTCACCTGC-TTTTT-3′ for shSK1#3.

In brief, 500 000 cells were seeded in T25 cell culture flasks. The plasmids were diluted in OptiMEM (Thermofisher) medium without serum. Cells were transfected with 5 μg shRNA oligomer using Lipofectamine 2000 reagent (Invitrogen) according to the manufacturer's instructions. Transfected cells were selected with 0.75 mg/ml G418 and 2 μg/ml puromycin and GFP-expressing cells were sorted by FACS. Stable transfectants were maintained in media containing 1 μg/ml puromycin; for the experiments, cells were cultured in medium without puromycin.

COLO829 were transfected with pcDNA3.1 or pcDNA3.1-SK1 plasmid (kindly obtained from Dr. SM. Pitson, Centre for Cancer Biology, Australia) and stable transfectants were selected for their resistance to 0.75 mg/ml G418 (Sigma).

Transient RNA interference was achieved by using a pool of four small interfering RNAs (siRNA) specific for S1PR1 (ON-TARGETplus SMARTpool; Dharmacon) or scrambled siRNA. Cells were transfected using HiPerfect Reagent (Qiagen) and inhibition efficiency was evaluated 48 h after transfection by Western blot. Monoclonal anti-EDG1 (S1PR1) and polyclonal anti-b-actin were purchased from Abcam and Cell Signaling, respectively.

### *In vitro* motility assay

Transmembrane cell migration assays were performed using Boyden chambers containing membranes with a pore size of 8 μm (Corning). One million cells of BMDM or THP-1 cells were suspended in serum-free DMEM in the presence or not of 2 μM VPC23019 (Coger, Paris, France), 5 μM W146 (Sigma) or 2 μM FTY720 (Sigma), and then added on top of the inserts. The bottom chamber was filled with CM from melanoma cells containing or not S1P (Biovalley, Nanterre, France) or serum-free medium either alone or containing S1P at the indicated concentrations. After the indicated time, macrophages that migrated to the underside of the insert membranes were harvested and quantified using a Coulter cell counter (Beckman).

### Quantitative RT-PCR

RNA from cells or tumors was extracted (RNeasy kit, Qiagen) according to the manufacturer's protocol and treated with RNase-free DNase (Qiagen). RNA quality was assessed by automated gel electrophoresis (Experion, BioRad). One μg of RNA was reverse transcribed (SuperScript II, Invitrogen) and the cDNA used as a template for qPCR. The reactions were performed in duplicate on the StepOne instrument (Applied Biosystems) using SYBR Green PCR kit and primer assay (QuantiTect, Qiagen). Results were quantified using the StepOne system software. mRNA of 18S and b-actin were analyzed for normalization. All the primers used were listed in Supplementary Table S1.

### Analysis of lipids

Lipids were extracted from tumors 10 days after melanoma cell implantation, and sphingolipids were quantified by ultra-performance liquid chromatography, using an Agilent 1290 system coupled to a G6460 triple quadripole spectrometer (Agilent Technologies) [45]. Alternatively, the amount of extracellular S1P was evaluated as reported [46] after incubation of the cells with 0.45 μCi/ml, 1.5 μM D-erythro-[3-^3^H] sphingosine (Perkin-Elmer).

### SK1 enzymatic assay

SK1 activity was determined as described [47] with minor modifications.

### ELISA for mouse TGF-β1

Murine TGFβ-1 from cell-free B16-F10 supernatants was measured by ELISA kit (Abcam) following the manufacturer's instructions.

### Bioinformatics

SK1 and TGF-β1 mRNA expression levels were assessed in melanoma patients of the Cancer Genome Atlas (TCGA) Skin Cutaneous Melanoma (SKCM) cohort (*n* = 472). Level 3 data was interpreted using gene transcription estimates as in RSEM normalized and centered log2 counts (https://genome-cancer.ucsc.edu/). Expression levels were correlated using Pearson product-moment correlation.

### Orthotopic melanoma grafts in mice

Animal experiments were conducted in accordance with national and international policies, and our protocol was approved by the Regional Ethics Committee of Midi-Pyrénées. shCtrl or shSK1 B16F10 cells (3.10^5^) were intradermally injected into the flank of 8-week-old C57BL/6 mice (Charles River, L'Arbresle, France). In some experiments, TGF-b-neutralizing antibody (1D11, Bio X Cell, 10 mg/kg) or PBS were *i.p.* administered three times per week starting one day after tumor cell injection and during 2 weeks.

To assess the contribution of macrophages to tumor growth, macrophages were depleted by *i.p*. administration of 200 μl of Liposomal Clodronate (Encapsula NanoSciences LLC) the day before tumor cell injection and then every 4 days for 2 weeks, as reported [48].

Tumor volume was calculated using a caliper at the indicated days as described [12]. Alternatively, tumors were removed at the indicated time after injection and weighed.

### Analysis of leukocyte content in tumors

B16F10 cells (3.10^5^) were intradermally injected into C57BL/6 mice. At day 10, mice were sacrificed and tumors were collected. Cells were counted and stained with the indicated antibodies and live-dead reactive dyes (Invitrogen) prior to flow cytometry analysis (BD LSRFortessa) [49]. Analyses were restricted to viable cells and performed using anti-CD45 (BD Biosciences, BUV395), anti-F4/80 (eBioscience, APC), anti-MHC-II (eBioscience, APC-e780), anti-CD206 (Biolegend, FITC) or anti-iNOS (Biolegend, PE-Cy7) antibody. Isotype controls were from Biolegend or eBioscience.

### Statistical analyses

Results are expressed as means ± sem, and group comparisons were performed with an unpaired two-tailed Student's *t* test for comparison of 2 groups, or one-way ANOVA followed by the post hoc Tukey test for comparison of experiments that consisted of ≥ 3 groups. The Mann-Whitney *U*-test was used to test statistical significance of differences in mean tumor growth between independent groups after treatment. Spearman's rank correlation test was used to determine the correlations of tumor size (Prism 6; GraphPad Software Inc, San Diego, CA). Pearson correlation was performed using GraphPad Prism. A *p*-value less than 0.05 was considered statistically significant (*, *p* < 0.05; **, *p* < 0.01; ***, *p* < 0.001).

## SUPPLEMENTARY MATERIALS FIGURES AND TABLE



## References

[1] Gajewski TF, Schreiber H, Fu YX (2013). Innate and adaptive immune cells in the tumor microenvironment. Nat Immunol.

[2] Brocker EB, Zwadlo G, Holzmann B, Macher E, Sorg C (1988). Inflammatory cell infiltrates in human melanoma at different stages of tumor progression. Int J Cancer.

[3] Jensen TO, Schmidt H, Moller HJ, Hoyer M, Maniecki MB, Sjoegren P, Christensen IJ, Steiniche T (2009). Macrophage markers in serum and tumor have prognostic impact in American Joint Committee on Cancer stage I/II melanoma. J Clin Oncol.

[4] Pollard JW (2004). Tumour-educated macrophages promote tumour progression and metastasis. Nat Rev Cancer.

[5] Gabrilovich DI, Ostrand-Rosenberg S, Bronte V (2012). Coordinated regulation of myeloid cells by tumours. Nat Rev Immunol.

[6] Payne AS, Cornelius LA (2002). The role of chemokines in melanoma tumor growth and metastasis. J Invest Dermatol.

[7] Wang T, Ge Y, Xiao M, Lopez-Coral A, Azuma R, Somasundaram R, Zhang G, Wei Z, Xu X, Rauscher FJ, Herlyn M, Kaufman RE (2012). Melanoma-derived conditioned media efficiently induce the differentiation of monocytes to macrophages that display a highly invasive gene signature. Pigment Cell Melanoma Res.

[8] Mantovani A, Sozzani S, Locati M, Allavena P, Sica A (2002). Macrophage polarization: tumor-associated macrophages as a paradigm for polarized M2 mononuclear phagocytes. Trends Immunol.

[9] Biswas SK, Mantovani A (2010). Macrophage plasticity and interaction with lymphocyte subsets: cancer as a paradigm. Nat Immunol.

[10] Wang T, Xiao M, Ge Y, Krepler C, Belser E, Lopez-Coral A, Xu X, Zhang G, Azuma R, Liu Q, Liu R, Li L, Amaravadi RK (2015). BRAF Inhibition Stimulates Melanoma-Associated Macrophages to Drive Tumor Growth. Clin Cancer Res.

[11] Colie S, Van Veldhoven PP, Kedjouar B, Bedia C, Albinet V, Sorli SC, Garcia V, Djavaheri-Mergny M, Bauvy C, Codogno P, Levade T, Andrieu-Abadie N (2009). Disruption of sphingosine 1-phosphate lyase confers resistance to chemotherapy and promotes oncogenesis through Bcl-2/Bcl-xL upregulation. Cancer Res.

[12] Albinet V, Bats ML, Huwiler A, Rochaix P, Chevreau C, Segui B, Levade T, Andrieu-Abadie N (2014). Dual role of sphingosine kinase-1 in promoting the differentiation of dermal fibroblasts and the dissemination of melanoma cells. Oncogene.

[13] Huwiler A, Pfeilschifter J (2008). New players on the center stage: sphingosine 1-phosphate and its receptors as drug targets. Biochem Pharmacol.

[14] Maceyka M, Harikumar KB, Milstien S, Spiegel S (2012). Sphingosine-1-phosphate signaling and its role in disease. Trends Cell Biol.

[15] LaMontagne K, Littlewood-Evans A, Schnell C, O'Reilly T, Wyder L, Sanchez T, Probst B, Butler J, Wood A, Liau G, Billy E, Theuer A, Hla T (2006). Antagonism of sphingosine-1-phosphate receptors by FTY720 inhibits angiogenesis and tumor vascularization. Cancer Res.

[16] Kunkel GT, Maceyka M, Milstien S, Spiegel S (2013). Targeting the sphingosine-1-phosphate axis in cancer, inflammation and beyond. Nat Rev Drug Discov.

[17] Deng J, Liu Y, Lee H, Herrmann A, Zhang W, Zhang C, Shen S, Priceman SJ, Kujawski M, Pal SK, Raubitschek A, Hoon DS, Forman S (2012). S1PR1-STAT3 signaling is crucial for myeloid cell colonization at future metastatic sites. Cancer Cell.

[18] Liang J, Nagahashi M, Kim EY, Harikumar KB, Yamada A, Huang WC, Hait NC, Allegood JC, Price MM, Avni D, Takabe K, Kordula T, Milstien S (2013). Sphingosine-1-phosphate links persistent STAT3 activation, chronic intestinal inflammation, and development of colitis-associated cancer. Cancer Cell.

[19] Dunn GP, Old LJ, Schreiber RD (2004). The immunobiology of cancer immunosurveillance and immunoediting. Immunity.

[20] Yang L, Han Z, Tian L, Mai P, Zhang Y, Wang L, Li L (2015). Sphingosine 1-Phosphate Receptor 2 and 3 Mediate Bone Marrow-Derived Monocyte/Macrophage Motility in Cholestatic Liver Injury in Mice. Sci Rep.

[21] Huang LS, Berdyshev E, Mathew B, Fu P, Gorshkova IA, He D, Ma W, Noth I, Ma SF, Pendyala S, Reddy SP, Zhou T, Zhang W (2013). Targeting sphingosine kinase 1 attenuates bleomycin-induced pulmonary fibrosis. Faseb J.

[22] Sorrentino R, Bertolino A, Terlizzi M, Iacono VM, Maiolino P, Cirino G, Roviezzo F, Pinto A (2015). B cell depletion increases sphingosine-1-phosphate-dependent airway inflammation in mice. Am J Respir Cell Mol Biol.

[23] Hussein MR (2006). Tumour-associated macrophages and melanoma tumourigenesis: integrating the complexity. Int J Exp Pathol.

[24] Colombo MP, Mantovani A (2005). Targeting myelomonocytic cells to revert inflammation-dependent cancer promotion. Cancer Res.

[25] Zeisberger SM, Odermatt B, Marty C, Zehnder-Fjallman AH, Ballmer-Hofer K, Schwendener RA (2006). Clodronate-liposome-mediated depletion of tumour-associated macrophages: a new and highly effective antiangiogenic therapy approach. Br J Cancer.

[26] Gude DR, Alvarez SE, Paugh SW, Mitra P, Yu J, Griffiths R, Barbour SE, Milstien S, Spiegel S (2008). Apoptosis induces expression of sphingosine kinase 1 to release sphingosine-1-phosphate as a “come-and-get-me” signal. Faseb J.

[27] Theilmeier G, Schmidt C, Herrmann J, Keul P, Schafers M, Herrgott I, Mersmann J, Larmann J, Hermann S, Stypmann J, Schober O, Hildebrand R, Schulz R (2006). High-density lipoproteins and their constituent, sphingosine-1-phosphate, directly protect the heart against ischemia/reperfusion injury *in vivo* via the S1P3 lysophospholipid receptor. Circulation.

[28] Zhang Z, Zhang ZY, Fauser U, Schluesener HJ (2008). FTY720 ameliorates experimental autoimmune neuritis by inhibition of lymphocyte and monocyte infiltration into peripheral nerves. Exp Neurol.

[29] Weigert A, Schiffmann S, Sekar D, Ley S, Menrad H, Werno C, Grosch S, Geisslinger G, Brune B (2009). Sphingosine kinase 2 deficient tumor xenografts show impaired growth and fail to polarize macrophages towards an anti-inflammatory phenotype. Int J Cancer.

[30] Murray PJ, Wynn TA (2011). Protective and pathogenic functions of macrophage subsets. Nat Rev Immunol.

[31] Mantovani A, Sica A, Sozzani S, Allavena P, Vecchi A, Locati M (2004). The chemokine system in diverse forms of macrophage activation and polarization. Trends Immunol.

[32] Hughes JE, Srinivasan S, Lynch KR, Proia RL, Ferdek P, Hedrick CC (2008). Sphingosine-1-phosphate induces an antiinflammatory phenotype in macrophages. Circ Res.

[33] Park SJ, Lee KP, Kang S, Lee J, Sato K, Chung HY, Okajima F, Im DS (2014). Sphingosine 1-phosphate induced anti-atherogenic and atheroprotective M2 macrophage polarization through IL-4. Cell Signal.

[34] Miller AV, Alvarez SE, Spiegel S, Lebman DA (2008). Sphingosine kinases and sphingosine-1-phosphate are critical for transforming growth factor beta-induced extracellular signal-regulated kinase 1 and 2 activation and promotion of migration and invasion of esophageal cancer cells. Mol Cell Biol.

[35] Ader I, Brizuela L, Bouquerel P, Malavaud B, Cuvillier O (2008). Sphingosine kinase 1: a new modulator of hypoxia inducible factor 1alpha during hypoxia in human cancer cells. Cancer Res.

[36] Alvarez SE, Harikumar KB, Hait NC, Allegood J, Strub GM, Kim EY, Maceyka M, Jiang H, Luo C, Kordula T, Milstien S, Spiegel S (2010). Sphingosine-1-phosphate is a missing cofactor for the E3 ubiquitin ligase TRAF2. Nature.

[37] Lin W, Tsai WL, Shao RX, Wu G, Peng LF, Barlow LL, Chung WJ, Zhang L, Zhao H, Jang JY, Chung RT (2010). Hepatitis C virus regulates transforming growth factor beta1 production through the generation of reactive oxygen species in a nuclear factor kappaB-dependent manner. Gastroenterology.

[38] Hung SP, Yang MH, Tseng KF, Lee OK (2013). Hypoxia-induced secretion of TGF-beta1 in mesenchymal stem cell promotes breast cancer cell progression. Cell Transplant.

[39] Busse A, Keilholz U (2011). Role of TGF-beta in melanoma. Curr Pharm Biotechnol.

[40] Wrzesinski SH, Wan YY, Flavell RA (2007). Transforming growth factor-beta and the immune response: implications for anticancer therapy. Clin Cancer Res.

[41] Malyshev I, Malyshev Y (2015). Current Concept and Update of the Macrophage Plasticity Concept: Intracellular Mechanisms of Reprogramming and M3 Macrophage “Switch” Phenotype. Biomed Res Int.

[42] Wojtowicz-Praga S, Verma UN, Wakefield L, Esteban JM, Hartmann D, Mazumder A (1996). Modulation of B16 melanoma growth and metastasis by anti-transforming growth factor beta antibody and interleukin-2. J Immunother Emphasis Tumor Immunol.

[43] Tas F, Karabulut S, Yasasever CT, Duranyildiz D (2014). Serum transforming growth factor-beta 1 (TGF-beta1) levels have diagnostic, predictive, and possible prognostic roles in patients with melanoma. Tumour Biol.

[44] Morris JC, Tan AR, Olencki TE, Shapiro GI, Dezube BJ, Reiss M, Hsu FJ, Berzofsky JA, Lawrence DP (2014). Phase I study of GC1008 (fresolimumab): a human anti-transforming growth factor-beta (TGFbeta) monoclonal antibody in patients with advanced malignant melanoma or renal cell carcinoma. PLoS One.

[45] Sorli SC, Colie S, Albinet V, Dubrac A, Touriol C, Guilbaud N, Bedia C, Fabrias G, Casas J, Segui B, Levade T, Andrieu-Abadie N (2013). The nonlysosomal beta-glucosidase GBA2 promotes endoplasmic reticulum stress and impairs tumorigenicity of human melanoma cells. FASEB J.

[46] Mitra P, Payne SG, Milstien S, Spiegel S (2007). A rapid and sensitive method to measure secretion of sphingosine-1-phosphate. Methods Enzymol.

[47] Lavieu G, Scarlatti F, Sala G, Carpentier S, Levade T, Ghidoni R, Botti J, Codogno P (2008). Sphingolipids in macroautophagy. Methods Mol Biol.

[48] van Rooijen N, Hendrikx E (2010). Liposomes for specific depletion of macrophages from organs and tissues. Methods Mol Biol.

[49] Bertrand F, Rochotte J, Colacios C, Montfort A, Tilkin-Mariame AF, Touriol C, Rochaix P, Lajoie-Mazenc I, Andrieu-Abadie N, Levade T, Benoist H, Segui B (2015). Blocking Tumor Necrosis Factor alpha Enhances CD8 T-cell-Dependent Immunity in Experimental Melanoma. Cancer Res.

