# Supplementary material for: Downregulation of sphingosine kinase-1 induces protective tumor immunity by promoting M1 macrophage response in melanoma
Source: Oncotarget. 2016 Sep 30;7(44):71873–86. doi: 10.18632/oncotarget.12380 (PMC5342129; doi:10.18632/oncotarget.12380)
Supplement: Supplementary file 1 [file oncotarget-07-71873-s001.pdf]

Downregulation of sphingosine kinase-1 induces protective tumor immunity by promoting M1 macrophage response in melanoma

Supplementary Materials

SUPPLEMENTARY MATERIALS AND METHODS

Analysis of leukocyte content in tumors

B16F10 cells (3.10^5) were intradermally injected into C57BL/6 mice. At day 10, mice were sacrificed and tumors were collected. Cells were counted and stained with the indicated antibodies and live-dead reactive dyes (Invitrogen) prior to flow cytometry analysis (BD LSRFortessa) [23]. Analyses were restricted to viable cells and performed using anti-Gr1 (Biolegend, PE), anti-CD11b (ebioscince, PE-Cy7), anti-Thy1 (Biolegend, APC-Cy7), anti-CD19 (BD Bioscience, APC), anti-NK1.1 (BD Bioscience, PE), anti-CD8 (Biolegend, BV605) or anti-CD4 (eBioscience, eFluor 450) antibody. Isotype controls were from Biolegend or eBioscience.

Cell proliferation

80 000 cells (B16F10 shCtrl or shSK1) were seeded in 6-well plates. 24, 48 and 72 hours later, cell proliferation was assessed by counting the trypsinized cells with a Beckman Coulter Counter.

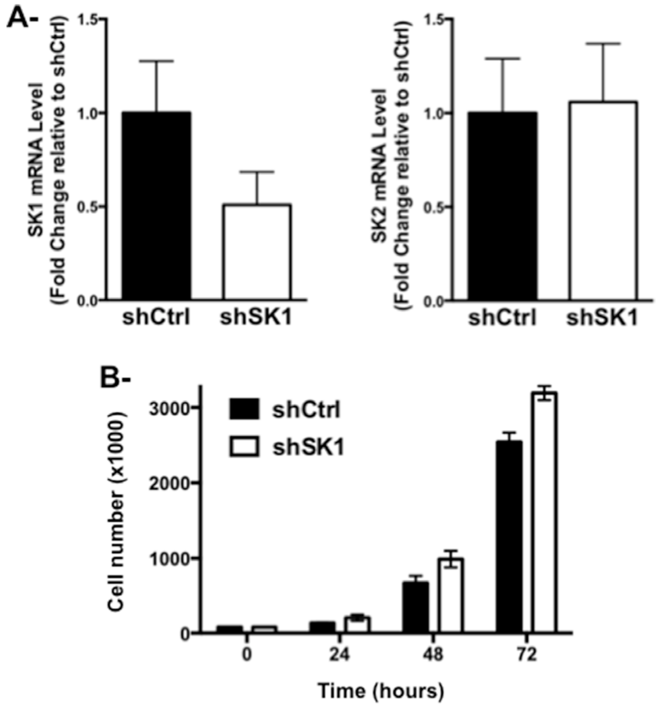

Supplementary Figure S1: Downregulation of SK1 in melanoma cells does not modify SK2 expression nor cell proliferation. (**A**) SK1 (left panel) and SK2 (right panel) mRNA levels were measured in B16F10 cells stably transfected with a control (shCtrl) or SK1 targeted shRNA (shSK1). Data are expressed as fold-change over shCtrl B16F10 cells and are means ± sem of 2 independent experiments. (**B**) Cell proliferation was evaluated by cell counting. Results represent means of 2 independent experiments.

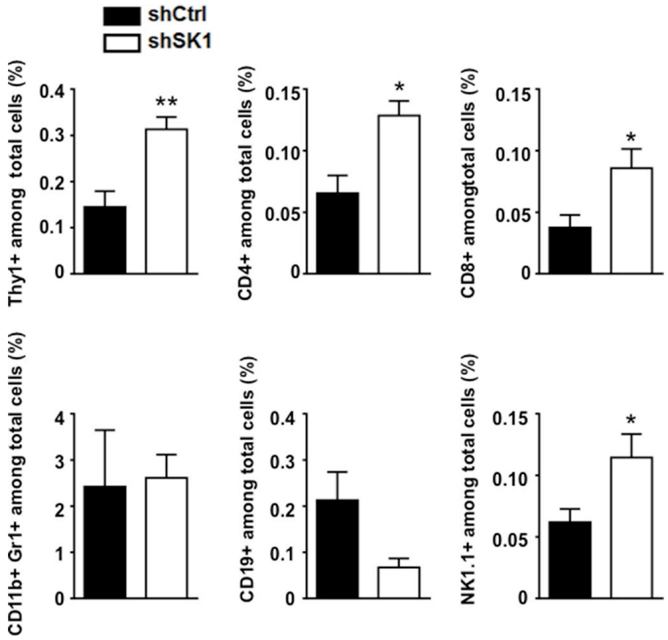

Supplementary Figure S2: Downregulation of SK1 in melanoma cells increases the number of intratumoral T-lymphocytes. shCtrl or shSK1 B16F10 murine melanoma cells were injected in C57BL/6 mice. Ten days after injection, mice were sacrificed and tumors were collected. Then, cells were counted and the proportion of the indicated cell populations was determined by flow cytometry. Top panel: Values indicate the percentages of $Thy1^+$ (left panel), $CD4^+$ (middle panel) and $CD8^+$ (right panel) cells among total cells. Bottom panel: Values indicate the percentages of $CD11b^+Gr1^+$ (left panel), $CD19^+$ (middle panel) and $NK1.1^+$ (right panel) cells among total cells. Bars represent means ± sem of 4 mice per group. Data are representative of two independent experiments.

Supplementary Table S1: Quantitative RT-PCR

Primer	Sequence or Reference	Supplier
Human β-actin	Hs_ACTB_1_SG QuantiTect Primer Assay QT00095431	Qiagen
Human SK1	Hs_SPHK1_1_SG QuantiTect Primer Assay QT01011927	Qiagen
Murine β-actin	ACTB forward: 5′ ACCTTCTACAATGAGCTGCG 3′	IDT DNA
	ACTB reverse: 5′ CTGGATGGCTACGTACATGG 3′	
Murine 18 S	18S forward: 5′ AGCCTGCGGCTTAATTTGAC 3′	IDT DNA
	18S reverse: 5′ CAACTAAGAACGGCCATGCA 3′	
Murine SK1	Mm_Sphk1_1_SG QuantiTect Primer Assay QT01046395	Qiagen
Murine YM1	YM-1 forward: 5′ CTTCCACAGGAGCAGGAATC 3′	IDT DNA
	YM-1 reverse: 5′ GCTCCATGGTCCTTCCAGTA 3′	
Murine ARG-1	ARG forward: 5′ AAGAATGGAAGAGTCAGTGTGG 3′	IDT DNA
	ARG reverse: 5′ GGGAGTGTTGATGTCAGTGTG 3′	
Murine iNOS	iNOS forward: 5′ CAGCTGGGCTGTACAAACCTT 3′	IDT DNA
	iNOS reverse: 5′ CATTGGAAGTGAAGCGTTTCG 3′	
Murine IL12	IL12p40 forward: 5′ CTGGCCAGTACACCTGCCAC 3′	IDT DNA
	IL12p40 reverse: 5′ GTGCTTCCAACGCCAGTTCA 3′	
Murine CD206	CD206 forward: 5′ CAAGGAAGGTTGGCATTTGT 3′	IDT DNA
	CD206 reverse: 5′ CCTTTCAGTCCTTTGCAAGC 3′	
Murine TNFα	Mm_Tnf_1_SG QuantiTect Primer Assay QT00104006	Qiagen
Murine IFNγ	Mm_Ifng_1_SG QuantiTect Primer Assay QT01038821	Qiagen
Murine CCL5	Mm_Ccl5_2_SG QuantiTect Primer Assay QT01747165	Qiagen
Murine CXCL9	Mm_Cxcl9_1_SG QuantiTect Primer Assay QT00097062	Qiagen
Murine CXCL10	Mm_Cxcl10_1_SG QuantiTect Primer Assay QT00093436	Qiagen
Murine TGFβ	Mm_Tgfb1_1_SG QuantiTect Primer Assay QT00145250	Qiagen
